# Impact of environmental comfort on urban vitality in small and medium-sized cities: A case study of Wuxi County in Chongqing, China

**DOI:** 10.3389/fpubh.2023.1131630

**Published:** 2023-02-06

**Authors:** Gaoxiang Liu, Jie Lei, Hongqiao Qin, Jiaqi Niu, Jianghua Chen, Jun Lu, Guifeng Han

**Affiliations:** ^1^College of Environmental and Ecology, Chongqing University, Chongqing, China; ^2^College of Architecture and Urban Planning, Chongqing University, Chongqing, China; ^3^School of Civil Engineering, Chongqing University, Chongqing, China

**Keywords:** environmental comfort, urban vitality, built-up areas, small and medium-sized cities, Wuxi County

## Abstract

China's urbanization has exceeded 64% and a large number of small and medium-sized cities are the key development areas in the new stage. In urban planning, it is very important to reveal the influence of environmental comfort on urban vitality to improve the life quality of residents in these towns. Thus, the study investigated the impact of environmental comfort on urban vitality using ordinary least squares regression in Wuxi County. Environmental comfort was assessed through a comprehensive analysis of a built-up area and urban vitality was represented by vitality intensity. In addition, the influence pathways were identified and model validation was verified. The conclusions are as follows: (1) Environmental comfort and urban vitality are distributed spatially similarly, and both gradually decline from the center to the periphery. It is high in the east and low in the west, high in the south and low in the north. (2) Population density, POI mixing degree, building density, and road network density have significant positive effects on urban vitality. Population density has the greatest impact on urban vitality. Building height, building age, and river buffer have significant negative effects on urban vitality. (3) The impact of comprehensive environmental comfort on urban vitality is positive, and in terms of time, the order of impact is afternoon > morning > evening. Finally, a method for assessing the impact of environmental comfort on urban vitality was constructed, and the promoting effect of environmental comfort improvements on the vitality was verified. These findings will fill the gap between urban physical space and social needs in planning practices and provide reference to improve vitality for urban planning in small and medium-sized cities.

## 1. Introduction

According to the United Nations, the urbanization rate of the developed countries will reach 86% in 2050, and that of China will reach 71.2% in 2050 (1–3). China's urbanization rate increased rapidly from 13.26% in 1953 to 64.72% in 2021 (4, 5), and the current rate is increasing at an annual rate of about 1% (6). There are about 20 thousand small and medium-sized towns, accounting for a significant portion of the population now. China is vigorously promoting the new urbanization plan and rural revitalization strategy. In the new urbanization strategy, the central government has set the tone to enhance the roles of small and medium-sized cities and small towns through industrial development, public services, employment absorption, and population agglomeration, which has opened up new opportunities and broad space for the development of small and medium-sized cities in China (7). Urban planning in the new stage should focus on the development of urban and rural spaces, improvements in urban quality, and the construction of an ecological civilization (8). The municipal land space planning guidelines clearly specify that the comfort and artistry of land space should be improved, that the quality and value of land space should be improved, and that improvements in urban quality and vitality should be included in urban renewal goals (9). China will invest much effort in the development of small and medium-sized cities in the future (10, 11). As the connections between villages and large cities, small and medium-sized cities and small towns can not only alleviate the pressure on the large cities but also promote the development of villages and the steady progress of new urbanization and rural revitalization strategies. However, relevant research on small and medium-sized cities is far more sparse than is research on large cities due to location disadvantages, poor geographical environments, and slow economic development in small and medium-sized cities. In the future, the advancement of new urbanization will gradually boost the development and construction of small and medium-sized cities, enhance environmental comfort, and promote the attractiveness of small and medium-sized cities. Therefore, the study of small and medium-sized cities is of great significance to rural revitalization as well as territorial spatial planning.

Early studies on environmental comfort mainly emphasized the comfort of natural climatic environments on a macro scale (12, 13). Then research gradually focused on more specific climate factors including sound, light, and heat (14–22). Most current studies have focused on the comprehensive evaluation of the natural, the built, and the social environment (23, 24). Clark examined the effect of environmental comfort on urban population growth along four dimensions: natural environmental comfort (climate, overall natural attractiveness, etc.), built environment comfort (large institutions, small firms, etc.), socioeconomic composition and diversity (residents' income, education, etc.), and residents' values and attitudes (friendly, hostile, etc.), and found that counties with high environmental comfort had higher growth rates (23). The relevant studies involved the environmental comfort of mesoscale spaces, such as residential (25) and commercial areas (26), and the environmental comfort of microscale interior spaces of buildings, such as office and residential buildings (27). This study will also evaluate environmental comfort in terms of the three aspects mentioned above, and these comfort features may enhance the attractiveness of a specific place.

From the general perspective of urban sociology, urban vitality is composed of economic vitality, social vitality, and cultural vitality, and urban vitality is only a spatial representation of economic, social, and cultural activities. In contrast, from an architecture and urban planning perspective, urban vitality is more people-driven. Jacobs (28) and Lewis Mundford et al. (29) pointed out that the urban spatial environment has a significant impact on human behavioral activities and that a dense population is a key element contributing to the vitality of public spaces (30). Urban vitality can be created through design (31). Urban vitality is directly and closely related to users, places, and activities (32) and is simultaneously affected by multiple environmental factors (33, 34). Many studies use pedestrian flow and attendance rates to represent urban vitality (35). Current studies on the impact of environmental comfort on urban vitality mainly focus on the impact of thermal comfort on urban vitality (36–39), and some studies also consider auditory and visual quality. The effects of various environmental factors must be simultaneously studied to ensure the overall environmental comfort of built-up areas.

Previous studies pay more attention to big cities, however, there are a large number of small and medium-sized ones that should be focused. Meanwhile, the built environment is complex, and the influencing factors of urban vitality exist diversification. As urban vitalities mainly consist of human beings and the influencing factors involve all aspects of the environment, it is particularly important to combine qualitative and quantitative research methods. With the further promotion of new urbanization and rural revitalization, it is imperative to investigate the environmental comfort and urban vitality of small and medium-sized cities. There is urgency to investigate the environmental comfort and urban vitality of small and medium-sized cities.

This study collected environmental factors and data of urban vitality of a built-up area in Wuxi County and used regression analysis to conduct their correlation. The objectives were formulated: (1) investigate the temporal and spatial distribution of environmental comfort and urban vitality; (2) conduct the impact of the environmental comfort on urban vitality; and (3) identified and verified the main influencing factors in residential and commercial areas. This study is not only a beneficial attempt to combine qualitative and quantitative data but can also effectively make up for the shortcomings of existing research on environmental comfort.

## 2. Data and methods

### 2.1. Study area

**Figure 1:**
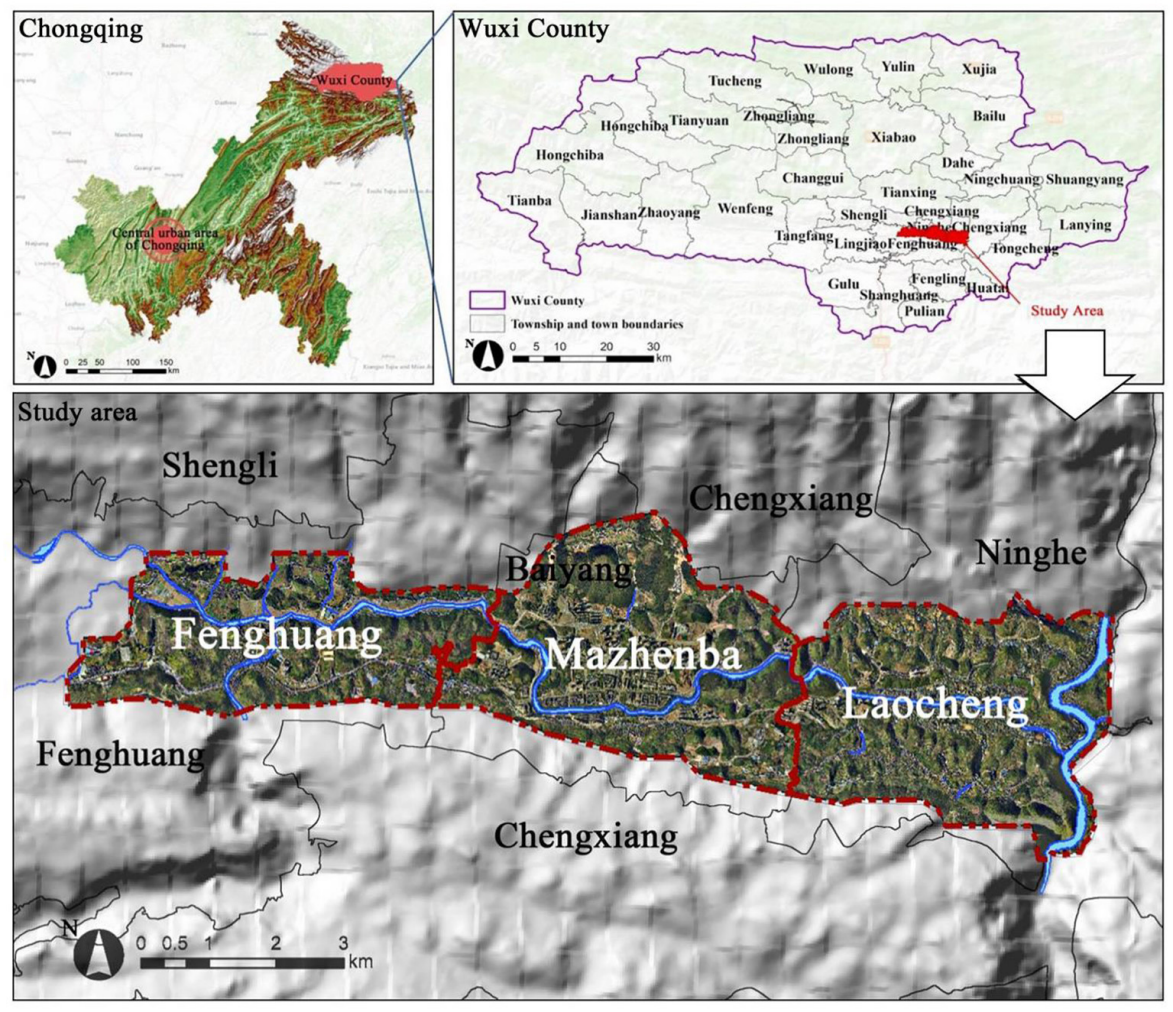
Location of the study area.

Wuxi is located in northeast of Chongqing with mountains surrounding in the west, is in a relatively closed state due to transportation and communication challenges. The influence of external factors recognized in existing studies on large cities can be excluded. The urban area of Wuxi is mostly composed of mountainous areas connected by the Baiyang River, forming a structure with three clusters in the city (Figure 1). Laocheng, Mazhenba, and Fenghuang, respectively represent small cities that are in the saturated development, accelerated and initial development period. In 2019, the built-up area of the city was 7.38 km^2^ with 115,600 resident persons. To facilitate the quantitative analysis and the establishment of a database, the study area was divided into 703 grid cells with a size of 200 m×200 m.

### 2.2. Data acquisition and processing

#### 2.2.1. Environment comfort

##### 2.2.1.1. Subjective environmental comfort factors

The indicators were constructed based on subjective and objective data. Subjective data were obtained through field surveys and questionnaire surveys to estimate natural, built and social environment by respondents. The Likert scale was used in this study to divide satisfaction into five levels (40), namely very satisfied (five points), relatively satisfied (four points), neutral (three points), relatively dissatisfied (two points), and very dissatisfied (one point) (Table 1). A total of 372 questionnaires were distributed, and 357 (96%) recovered questionnaires were valid.

**Table 1:** Subjective environmental comfort factors.

**Environment attributes**	**Environmental factors**	**Score**
Natural environment	Temperature and humidity	Very satisfied (five points) Relatively satisfied (four points) Neutral (three points) Relatively dissatisfied (two points) Very dissatisfied (one point)
	Natural ventilation	
	Air quality	
Built environment	Auditory environment	
	Visual environment	
	Green environment	
	Event venues	
	Transportation convenience	
Social environment	Safety	
	Overall evaluation	

##### 2.2.1.2. Objective environmental comfort factors

Thirteen environmental comfort factors were selected for objective environmental comfort in three categories (natural, built, and social environment) (Table 2). Data from: Wuxi County administrative boundary and water system layers from the National Center for Basic Geographic Information System; basic data from the processed third national land survey of Wuxi County; road network system integrated with the 2019 urban control plan of Wuxi County; satellite remote sensing image maps of Wuxi County; topographic survey current building vector data of Wuxi County in 2019 (containing the number of floors, shape, and other attributes of each building); Wuxi County 2019 Statistical Yearbook; relevant partial door data of Wuxi County. The calculation methods are as follows.

**Table 2:** Objective environmental comfort factors.

**Environmental factors**	**Factors**	**Description**	**Positive (P) or negative (N) effects**	**Weight coefficient**
Natural environment	River buffer zone (RBZ)	The further away from the river, the greater the value.	N	2.91%
		Slope (SL)	The higher the SL, the larger the value	N	0.82%
Built environment	Sky visibility (SV)	The higher the SV, the higher the value	P	0.36%
		Green coverage rate (GCR)	The higher the GCR, the higher the value	P	2.37%
		Road network density (RND)	The greater the RND, the greater the value	P	3.82%
		Comprehensive accessibility (CA)	The higher the CA, the higher the value	P	0.60%
		Building density (BD)	The greater the BD, the greater the value	N	0.52%
		Building height (BH)	The higher the BH, the larger the value	N	0.99%
		Building age (BA)	The newer the building, the higher the value	P	12.01%
		Density of public service facilities (PSF)	The greater the PSF, the greater the value	P	35.56%
		POI mixing degree (PMD)	The higher the PMD, the larger the value	P	14.52%
Social environment	Population density (PD)	The greater the PD, the greater the value	P	14.75%
		Per capita living area (PLA)	The larger the area, the larger the value	P	10.77%

a) Slope (SL). Using the ArcGIS 10.5 spatial analysis tool to analyze the elevation information of the research area, according to the suitability evaluation of urban construction land, the slope is divided into 5 grades, namely ≤10°, 10–15°, 15–20°, 20–25°, ≥25°, respectively represent “suitable construction area,” “more suitable construction area,” “basically suitable construction area,” “unsuitable construction area” and “non-construction area.”
b) River buffer zone (RBZ). The area covered by 100, 200, 300, 400 m and more than 400 m, based on the river's shoreline and extending outward, indicates the areas of high, higher, average, lower and low using the river's landscape resources respectively.
c) Sky visibility (SV). Firstly, the three-dimensional urban space environment of the research area is established. Then use the skyline and skyline map tools in the ArcGIS platform to calculate the sky visibility of a point in the three-dimensional space.
d) Green coverage rate (GCR). The green coverage rate was obtained by comparing the canopy density information in the second survey data of the Forestry Bureau, the park green space information of the Wuxi County Third National Land Survey, the park green space information of the current land use, and satellite images.
e) Road network density (RND). The road network selected in this study includes four categories: urban arterial roads, urban secondary arterial roads, urban branch roads, and urban pedestrian paths. Use the current road analysis map and satellite image map integrated in the 2019 regulatory detailed planning of Wuxi County to improve the current road network map. Calculate the ratio of the total road length to the area in each grid as the road network density value.
f) Comprehensive accessibility (CA). The average weighted travel time from the origin to all facilities. The walking mode is the main way, with each residential point as the starting point and the public service facility point as the destination point, and the walking time cost is set to 5km/h. Starting from starting point *i*, the average weighted travel time to all facilities *j* is Eq. 1, where *T*_*i*_ is the accessibility of starting point *i, p*_*ij*_ is the travel probability from starting point i to facility *j* (Equation 2); *t*_*ij*_ is the starting travel time from starting point *i* to facility point *j*; *m*_*j*_ is the area of facility *j*; a represents the attenuation coefficient, and *a* = 2.0 is selected in this paper; *d*_*ij*_ is the distance from starting point *i* to facility *j*. The lower the calculated value, the higher the accessibility, and the higher the value, the lower the accessibility.

(1) $$T_{i}=\sum_{\begin{array}{c} j=1\\ j\neq i \end{array}}^{n}(p_{ij}t_{ij})$$

(2) $$p_{ij}=\frac{m_{j}/d_{ij}^{a}}{\sum_{j}m_{j}/d_{ij}^{a}}$$

g) Building density (BD). In ArcGIS 10.5, the proportion of the building is calculated through the outer frame of the building, the mass points of the building are extracted, and the building density is calculated using the spatial kernel density analysis tool.
h) Building height (BH). Building height = number of building floors ^*^ 3, extract the mass points of the building in ArcGIS 10.5, and use the kernel density analysis tool in the spatial analysis to obtain the building height.
i) Building age (BA). The age of buildings is divided into five categories: before 1980, 1980–1990, 1990–2000, 2000–2010, 2010–2020, respectively assigned 1, 2, 3, 4, 5 points in ArcGIS 10.0.
j) Density of public service facilities (PSF). Using the ArcGIS 10.0 spatial network analysis tool, the service scope of public service facilities with different service levels is established, and the density of public service facilities is obtained.
k) POI mixing degree (PMD). By writing python code, the POI data of Gaode map and Baidu map POI data in the research area were crawled, a total of 8,668 items. The data was collected in December 2020. Through data screening and cleaning, the data outside the scope of the study area, as well as data with repeated coordinate points and repeated information were eliminated. Finally, a total of 7,548 pieces of data were obtained. All data have undergone coordinate correction, space correction and projection (CGCS2000). POI data is divided into 10 categories: government, transportation, business, enterprise, education, green space, residential, medical, sports, and others. Use the method of information entropy to calculate the POI mixing degree of each cell grid (Equation 3). It is used to represent the mixed degree of land use. The higher the calculated value, the better the mixed function of the plot.

(3) $$H\left(X\right)=-\sum_{i=1}^{N}Pi\;\log Pi$$

where *H(X)* is the entropy of random variable X; *Pi* is the probability of *X* taking *Xi*. *N* is the number of types of POI types, and *Pi* is the relative density of the *i*-th type of POI in the unit grid divided by the sum of the relative densities of various POIs in the unit grid.

l) Population density (PD). According to the data of Wuxi County Statistics Bureau, the population data of each street and each residential group in the study area are obtained. Extract a total of 536 current residential land plots in 2020 (including information such as plot area, plot ratio, building density, etc.), and calculate the average population density of the research area through the land area and plot ratio (Equation 4)

(4) $$RAPD=TP\times \frac{PPR\times PA}{CARL}\times \frac{1}{PA}$$

where *RAPD* is the residential area population density, *TP* is the township population, *PPR* is the plot ratio of the parcel, *PA* is the plot area, *CARL* is the total construction area of residential land

m) Per capita living area (PLA). First, all the buildings covered by the residential plot are calculated as residential buildings, and the residential buildings are extracted. Second, using the building as a particle, use Equation 5 to calculate the per capita living area. The resulting PLA was divided into five categories: >55 m^2^, 47–55 m^2^, 39–47 m^2^, 31–39 m^2^, and <30 m^2^. Respectively, the per capita living area is high, relatively high, normal, relatively low and low.

(5) $$PLA=\frac{\sum_{i=1}^{n}BA_{i}\times BS_{i}}{PURP}$$

where *BA*_*i*_ is the building area, *BS*_*i*_ is the Building Stories, *PURP* is the total number of residents in the unit.

The weight coefficient of each environmental indicator was calculated using the entropy method. First, the data were non-dimensionalized using range standardization to standardize the values of indicators with different measurement units and different properties. Assuming that *k* indicators (*X*_1_, *X*_2_,…, *X*_*k*_, where *X*_*i*_ = { *x*_1_, *x*_2_... *x*_n_}) are given, the formula is as follows which Equation 6 is applicable to indicators with positive impacts, and Equation 7 is applicable to indicators with negative impacts:


(6) $$X_{ij}^{{}^{\prime }}=\frac{X_{ij}-min[X_{j}]}{max\left[X_{j}\right]-min[X_{j}]}*100$$

(7) $$X_{ij}^{{}^{\prime }}=\frac{\max\left[X_{j}\right]-X_{ij}}{max\left[X_{j}\right]-min[X_{j}]}*100$$

where *i* = 1..., *n* (*n* is the number of grid); *j* = 1..., m (m is the number of indicators); *X*_*ij*_ represents the value of the *jth* indicator of the *ith* grid cell; max[*X*_*j*_] is the maximum value of the indicator series;[*X*_*j*_] is the minimum value of the indicator series; and $X_{ij}^{{}^{\prime }}$ is the standardized value of the *jth* indicator of the *ith* grid.

Second, the entropy method was used to determine the weights of environmental factors (41). The information entropy of each set of data was calculated using the definition of information entropy from information theory (Equation 8):


(8) $$E_{j}=-ln{\left(n\right)}^{-1}\sum_{i=1}^{n}[P_{ij}\;lnP_{ij}]$$

where $P_{ij}=X^{\prime }/\sum_{i=1}^{n}X_{ij}{}^{\prime }$ (if *P*_*ij*_ = 0, then *E*_*j*_ = 0); and n is the number of grid cells in the study.

The information entropy of each indicator could be calculated by Equation 9. Then, the weight of each indicator was calculated and the values are shown in Table 2:


(9) $$P_{ij}=\frac{1-E_{j}}{k-\sum_{j=1}^{k}E_{j}}\;$$

#### 2.2.2. Urban vitality data

The Tencent Easygo heat map records real-time geographic information left by users when they visit WeChat, QQ, and Tencent Maps, which are updated every 15 min. The number of users in each location can better reflect the population distribution characteristics in a specific area and time period and intuitively express urban vitality in an area. Real-time heat map data from 06:00 to 24:00 were crawled from the Tencent Easygo heat map (https://heat.qq.com/document.php). Python language were used to collect the real-time data through interface of Tencent Map. The data of August 14, 2020 (weekdays) and August 15, 2020 (weekends) were, respectively crawled with 1 h interval. A total of 38 groups of data (including time, longitude, dimension and population characteristics) were collected. All data are summarized to represent the comprehensive urban vitality of the study area. Using the spatial analysis tool, it is presented spatially and we can obtain the spatial distribution data of urban vitality in the study area. In terms of temporal distribution, the values of each time point are summarized form the data of the city's variation value to form urban vitality of each period (morning, afternoon, evening). Finally, we obtained the data with a size of 25 × 25 m, and all data are spatially corrected and projected (CGCS2000). And the values were averaged to a size of 200 × 200 m using ArcGIS10.5.

### 2.3. Methods

#### 2.3.1. Assessing environmental comfort

The subjective environmental indicators were evaluated using the average value. A multi-indicator comprehensive evaluation method (42) was used to evaluate objective environmental comfort in the following steps: (1) data standardization, (2) determination of the weights of environmental factors using the entropy method (41), and (3) calculation of environmental comfort using the multi-indicator comprehensive evaluation method. This research proposes a general algorithm: S = A^*^Qz+B^*^Qk (Qz: subjective; Qk: objective). Different cities may have different values of A and B due to various sizes, locations, and stages of economic development. Based on previous studies (43–45) and the actual situation, the values of A and B in this study were determined to be 0.3 and 0.7, respectively.

The above method was used to calculate the environmental comfort of a single grid, and then, ArcGIS 10.5 was used for the spatial visualization analysis. The natural breaks method was used to divide environmental comfort into five levels, namely high, relatively high, moderate, relatively low, and low.

#### 2.3.2. Analysis of urban vitality

The kernel density of the heat map data was calculated on the ArcGIS platform. After excluding values of 0 (areas with no vitality), the calculated heat map data were divided into five levels from high to low using the natural breaks method (46) including high-vitality, relatively high-vitality, moderate-vitality, relatively low-vitality, and low-vitality areas.

#### 2.3.3. Ordinary least squares (OLS) regression analysis

OLS regression analysis is a statistical analysis method used to determine the quantitative relationship between two or more variables. Environmental comfort involves many factors, the OLS method is a representative option for the regression analysis of environmental comfort. The bivariate calculation method for determining local indicators of spatial association (LISA), i.e., Moran's I statistic, was used to analyze the spatial relationship between environmental comfort and comprehensive urban vitality. Through SPSS 2021, a regression model was established based on qualitative and quantitative data, and the regression coefficients and fitted values were used to explain the impact of environmental comfort on urban vitality.

#### 2.3.4. Urban vitality enhancement in residential and commercial areas

Residential areas are the places most closely associated with people's lives, and commercial areas are highly common public spaces used by residents. Residential and commercial areas both account for a high proportion of urban land. Therefore, residential and commercial areas were selected to validate the underlying impact of environmental comfort on urban vitality.

The typical residential area on the north bank of the Baiyang River in Laocheng was constructed a long time ago. Therefore, the current facilities are relatively out of date, the spatial connectivity is poor, and the green space is scarce. The Yiboyuan community in Mazhenba is a modern residential area that is dominated by high-rise buildings, but the community is relatively closed and has poor connectivity to the outside world. The third and fourth communities of Shuangfeng Village are two concentrated residential areas in Fenghuang. All the case areas are close to rivers, and the surrounding construction land types are simple, which reduces additional impacts caused by the surrounding land.

Yuning Street and Nanzheng Street are the commercial centers in northern Laocheng. The commercial center mainly has modern commerce with the mostly commercial complexes buildings on the east side of Xiaoyao Square in Mazhenba. The study areas were all close to the river, and the surrounding land was mainly residential land. The main factors affecting the urban vitality of residential and commercial areas obtained by using correlation analysis and OLS regression, respectively, are first optimized for the current situation to verify their contribution to urban vitality. For convenience, the case areas were divided into 20 × 20 m grid cells.

## 3. Results and analysis

### 3.1. Characteristics of environmental comfort

**Figure 2:**
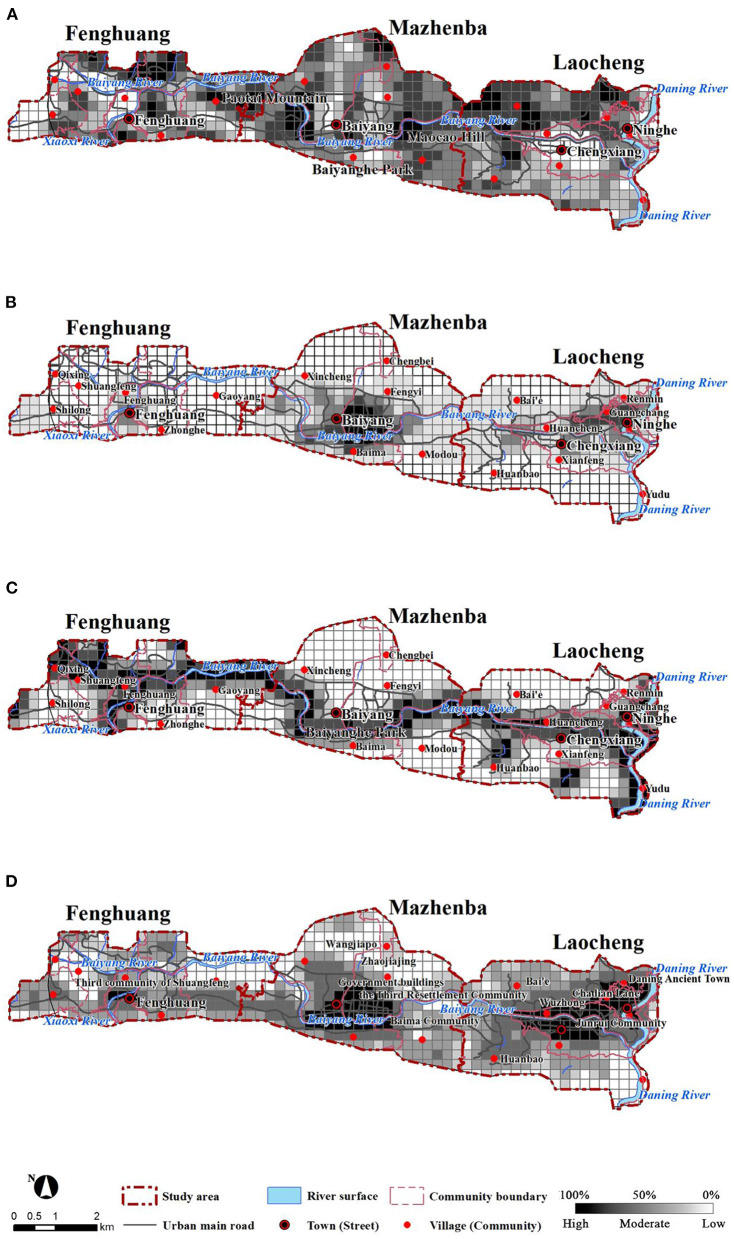
Distribution of environmental factors: green coverage rate **(A)**, density of public service facilities **(B)**, river buffer zones **(C)**, and comprehensive environmental comfort **(D)**.

In spatial distribution, the green coverage rate, building height, per capita living space, and road network density exhibited similar multicenter patterns (Figure 2A). The high values of public service facilities density, POI mixing degree, and population density were mainly existed in Laocheng and Mazhenba, with a single-center pattern (Figure 2B). However, the spatial distributions of rivers, slopes, and sky visibility were similar (Figure 2C). Green coverage rates were clustered toward multiple points, and those for roads, squares, and commercial land were lower than the surrounding environment. Public service facilities, which are most closely related to the daily life of residents, were mainly concentrated in the places where people gather, with a heterogeneous spatial distribution, and their density slowly decreased outwards with Mazhenba as the center. based on the spatial distribution and directions of the rivers, the river buffer zones relatively concentrated in Fenghuang and Laocheng.

The comprehensive environmental comfort is shown in Figure 2D. The spatial distribution pattern of environmental comfort was similar to public service facilities, road network density, comprehensive accessibility, and population density patterns. The overall comfort gradually decreased from east to west, and it was comparable in Laocheng and Mazhenba and lowest in Fenghuang.

Public service facilities and population density had great impacts on environmental comfort. High environmental comfort mainly concentrated in Mazhenba and Laocheng, which had the highest population densities, POI mixing degrees, and a large-scale public service facility. These features indicated that the comfort in Laocheng and Mazhenba was highest. Relatively high comfort concentrated in the central parts of the three clusters, including residential areas, public activity spaces, urban arterial roads, and spaces along the banks of the Daning River, Baiyang River, and Xiaoxi River. These areas basically cover the land for public service facilities (such as administrative offices, commercial services, healthcare, and education et al.) and show spatial correspondence with population density and public service facilities. Relatively low comfort mainly distributed at the southern end of the Daning River, Wangjiapo, and Shuangfeng Village, which are located relatively close to rivers. But the areas have greatly undulated terrain, large slopes, low population densities, a small number of residential areas, large land area, sparse population, few supporting facilities, and a lack of residential land and other facilities.

### 3.2. Characteristics of urban vitality

#### 3.2.1. Temporal characteristics

**Figure 3:**
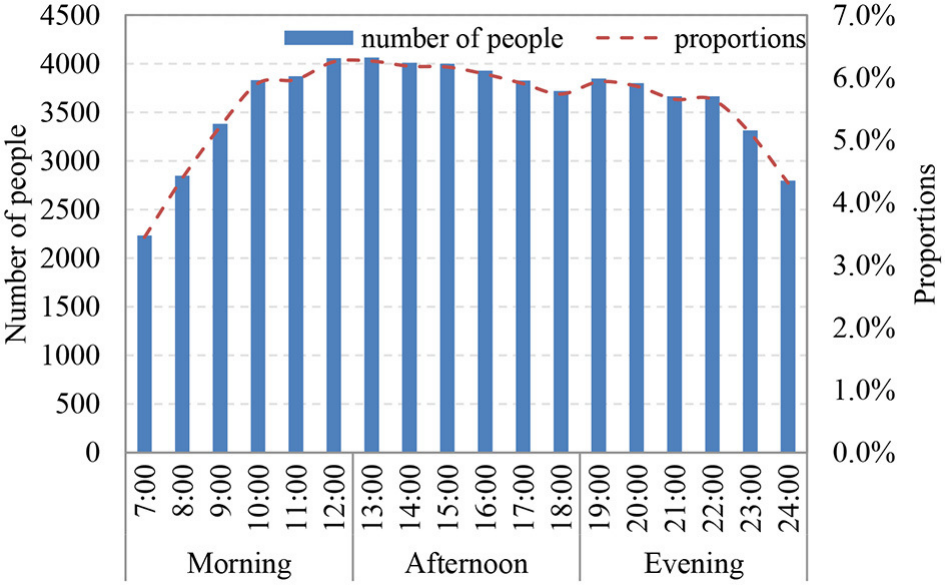
Changes in proportions of urban vitality from morning to evening.

Weekdays and weekends were divided into three time periods: morning (07:00–12:00), afternoon (13:00–18:00), and evening (19:00–24:00). It indicated that urban vitality was the highest in the afternoon (Figure 3). The morning total accounted for 31% of the day, and that for the afternoon and the evening were, respectively 36 and 33%.

From the morning to the afternoon, the population exhibited an inflow. In the morning, urban vitality changed rapidly. Activities changed from static activities to dynamic activities. The population mobility was large. The residents transitioned from rest to work or to leisure and entertainment. The type and scope of activities also changed significantly, and the number of people participating in urban activities increased. Among the surveyed population, 43% of the people worked and lived in various clusters, and 10% worked outside the urban area, indicating that from morning to afternoon, there was more crowd flow between the three clusters and a small population outflow at the same time.

Urban vitality declined from the afternoon to the evening, manifesting as a population outflow pattern. However, urban vitality in the evening (36%) was still higher than that in the morning (31%). The number of people who participated in activities in the evening was the highest, indicating that the facilities provided people with employment and various services. In the evening, the population flow was small, and the residents entered the rest state relatively late. The mismatch between workplaces and residence can be the part reason and the limited function of urban land use. For example, Mazhenba can provide many employment opportunities and rest and entertainment services, and Fenghuang can provide low-cost residential services.

#### 3.2.2. Spatial characteristics

**Figure 4:**
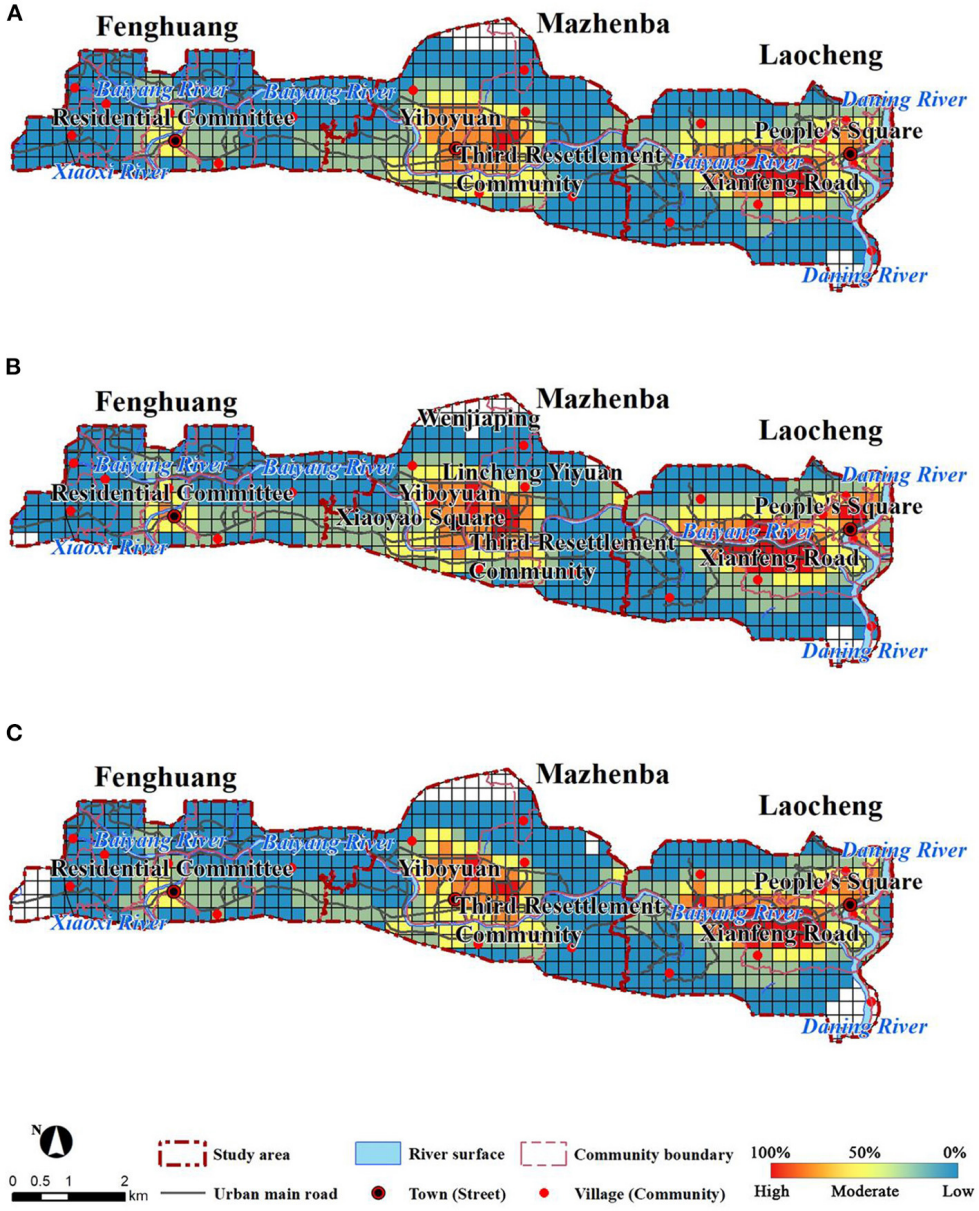
Distributions of urban vitality: morning **(A)**, afternoon **(B)**, and evening **(C)**.

The scope of vitality was the largest in the morning and the lowest in the evening (Figure 4). Vitality intensity was the highest in the afternoon and the lowest in the morning. In the morning, the high-vitality areas concentrated in Laocheng and Mazhenba, and the distributions of high vitality performed multiple points in the afternoon, Low-vitality areas concentrated in Fenghuang and the transition areas between the three clusters. In the afternoon, the scope of high-vitality areas gradually increased. There was high vitality in the people's square in the north of Laocheng, relatively high vitality in Fenghuang. The scope of low-vitality areas decreased, and the vitality between Mazhenba and Fenghuang increased. The distribution of vitality in the evening was similar to that in the morning, From the morning to the evening, areas with no vitality increased in Fenghuang, and the urban vitality decreased significantly in southern Mazhenba.

Population density, buildings, and various public service facilities (such as residential land such as the Yiboyuan) mainly distributed in the central parts of the three clusters. The urban functional areas were relatively consistent with urban vitality. The urban centers with abundant industries and complete public service facilities can satisfy most current needs, provide job opportunities on weekdays, and attract the surrounding residents for shopping, dining, leisure and entertainment on weekends. The activities of most residents concentrated in the urban centers. Therefore, urban vitality is high in urban centers. Fenghuang, an area with low vitality, has limited commercial and recreational facilities, with a large scope of vitality but a low overall vitality.

Due to the shortage of construction land, the burden on the urban centers was relatively large, but improvements in environmental comfort could be achieved by upgrading and improving existing spaces. The southern and northern marginal areas have relatively poor terrains, challenges with regard to development and construction, a low population density, and relatively low urban vitality. In Fenghuang, all types of facilities and supporting infrastructure are in the early stages of development and cannot provide more job opportunities on weekdays or leisure and entertainment facilities on weekends. Although Fengcheng has a large scope of vitality, its vitality is low due to the small spaces with high population agglomeration.

### 3.3. Relationship between environmental comfort and urban vitality spatial distribution

The bivariate calculation method for determining local indicators of spatial association (LISA), i.e., Moran's I statistic, was used. The analysis results were divided into five types: high-comfort and high-vitality (HH), high-comfort and low-vitality (HL), low-comfort and high-vitality (LH), low-comfort and low-vitality (LL), and non-significant (NS). The matching degree in the afternoon was better than that in the evening or morning (Table 3).

**Table 3:** Proportions of different matching types of environmental comfort and urban vitality.

**Types**	**Morning**	**Afternoon**	**Evening**
NS	46.09%	46.09%	46.09%
HL	2.28%	1.99%	2.13%
LH	1.42%	0.85%	1.00%
LL	33.71%	34.28%	34.57%
HH	16.50%	16.79%	16.22%

**Figure 5:**
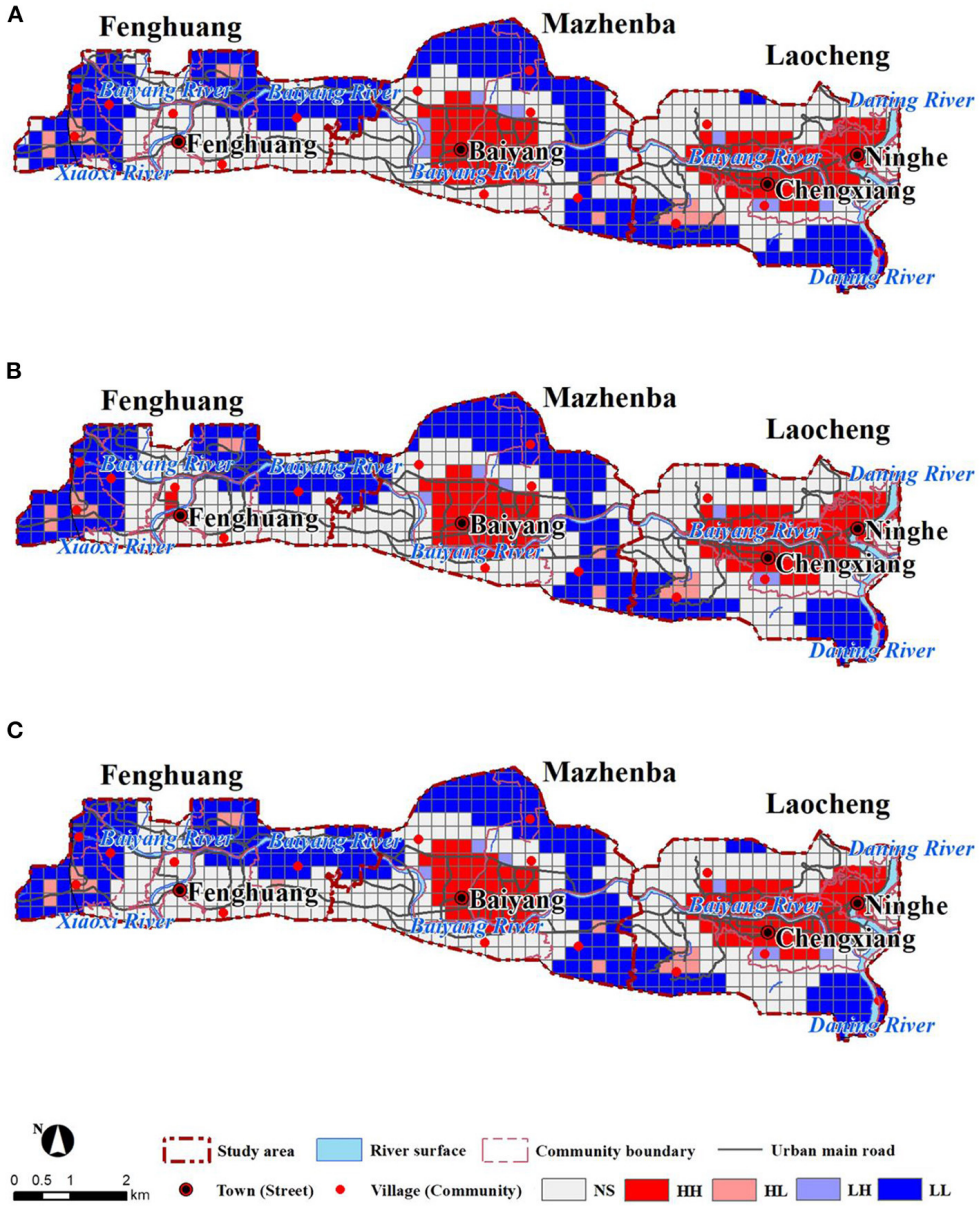
Spatial matching of environmental comfort and urban vitality: morning **(A)**, afternoon **(B)**, and evening **(C)**.

Spatially, the HH region contained Mazhenba and Laocheng as the center (Figure 5). From the center to out, there were HH, LH, NS, HL, and LL areas. Both Laocheng and Mazhenba exhibited obvious agglomeration, with a large proportion of HH and a smaller proportion of HL and LH zones, and a better spatial match between environmental comfort and urban vitality. In Fenghuang, NS and LL areas were dominant, and HL and LH areas accounted for a relatively large proportion. Overall, the spatial match between environmental comfort and urban vitality was high. HH and LL zones accounted for a higher proportion and were distributed in the central urban area and the urban fringe with better natural environment, respectively. The urban vitality in the central city is high and the environmental comfort is at a better level. HL and LL zones are less spatially distributed.

### 3.4. Impact of environmental comfort on urban vitality

#### 3.4.1. Correlation analysis

**Figure 6:**
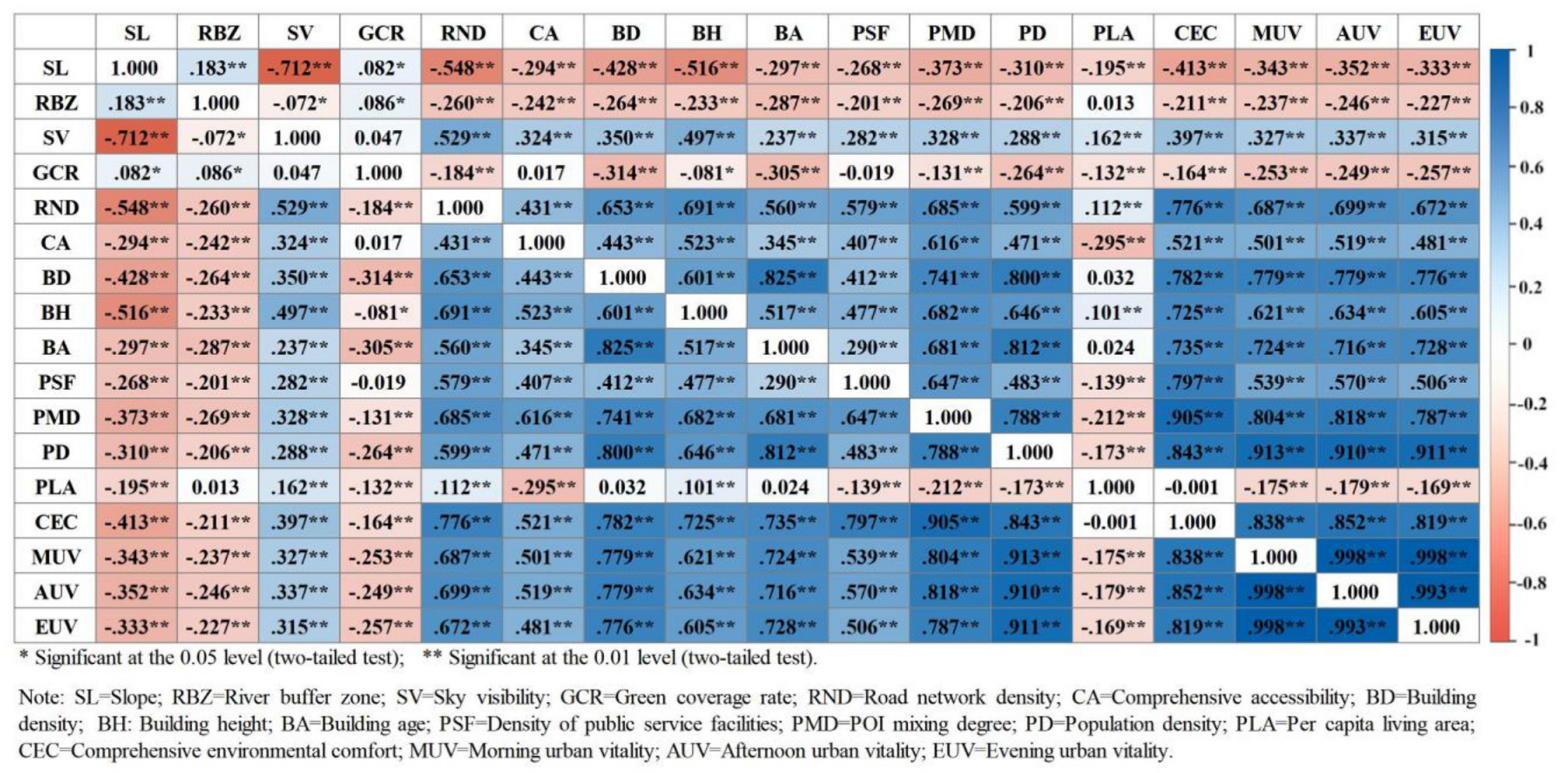
Pearson correlation between objective environmental comfort factors and urban vitality. *Significant at the 0.05 level (two-tailed test); **Significant at the 0.01 level (two-tailed test). SL, Slope; RBF, River buffer zone; SV, Sky visibility; GCR, Green coverage rate; RND, Road network density; CA, Comprehensive accessibility; BD, Building density; BH, Building height; PSF, Density of public service facilities; PMD, POI mixing degree; PLA, Per capita living area; CEC, Comprehensive environmental comfort; MUV, Morning urban vitality; AUV, Afternoon urban vitality; EUV, Evening urban vitality.

The correlations between the 13 factors and urban vitality were assessed be calculating Pearson correlation coefficients. Environmental factors and comfort were significantly correlated with urban vitality. Building density and building age were relatively highly correlated with population density, and comprehensive environmental comfort, population density, POI mixing degree, and building density were relatively highly correlated with urban vitality. The correlations between slope, river buffer zones, sky visibility, and per capita living area were low (Figure 6).

#### 3.4.2. Multivariate regression analysis

Based on the correlation results, stepwise OLS regression analysis was performed on the 13 factors. The results showed that the variance inflation factor (VIF) values of the 13 factors all <7.5, indicating that all factors can be included in the calculation. The 13 environmental factors were used as independent variables, and urban vitality in the morning, urban vitality in the afternoon, and urban vitality in the evening were used as dependent variables for the OLS regression analysis. Table 4 shows the OLS regression analysis results when different conditions are controlled. The *R*^2^ value for the model for urban vitality in the afternoon was 0.888, indicating that the 13 environmental factors explain 88.8% of the variance in urban vitality in the afternoon. Additionally, the degree of fit was better for the evening than for the morning and evening, indicating that the effect of overall factors on urban vitality was more significant.

**Table 4:** The OLS regression model between urban vitality and 13 environmental factors.

	**Urban vitality**		
	**Morning**	**Afternoon**	**Evening**
Slope	0.025	0.024	0.026
River buffer zone	−0.029^*^	−0.032^*^	−0.025
Sky visibility	0.015	0.017	0.012
Green coverage rate	−0.020	−0.024	−0.015
Road network density	0.227^**^	0.220^**^	0.233^**^
Comprehensive accessibility	0.019	0.029	0.009
Building density	0.096^**^	0.094^**^	0.097^**^
Building height	−0.113^**^	−0.102^**^	−0.124^**^
Building age	−0.172^**^	−0.176^**^	−0.166^*^
Density of public service facilities	−0.010	0.025	−0.046
POI mixing degree	0.132^**^	0.140^**^	0.123^**^
Population density	0.793^**^	0.792^**^	0.823^**^
Per capita living area	−0.018	−0.020	−0.017
*R* ^2^	0.881	0.888	0.871
Adjusted *R*^2^	0.879	0.886	0.868
*F*	394.235	422.273	357.595

* Significant at the 0.05 level (two-tailed test).

** Significant at the 0.01 level (two-tailed test).

The impact of environmental factors on urban vitality in the three time periods was consistent. Population density, POI mixing degree, building density, and road network density had significant positive impacts on urban vitality, and the increase in these four factors promoted urban vitality. The regression coefficients for population density were 0.793, 0.762, and 0.823 for the morning, afternoon, and evening, respectively. The impact of population density on urban vitality was greater in the evening than in the morning or afternoon, and population density had a greater impact on urban vitality than did other factors (that is, population density had the greatest impact on urban vitality). Road network density had the second highest impact on urban vitality, and the regression coefficients in the morning, afternoon, and evening were 0.227, 0.220, and 0.233, respectively, indicating that the impact was the largest in the evening. The impact of POI mixing degree on urban vitality was the greatest in the afternoon, and the impact of building density was the greatest in the morning. Areas with a high road network density, POI mixing rate, building density, and population density had high urban vitality. High values of these factors and urban vitality mainly concentrated in central areas with many squares and roads and high sky visibility, for example, public service facilities on the banks of the Baiyang River and the Shuiyuan Mall in the central areas. Building height, building age, and river buffer zones had significant negative impacts on urban vitality, indicating that taller buildings, newer buildings, and buildings farther away from rivers reduced urban vitality. New and high buildings and areas far away from rivers (such as the southern and northern urban areas and newly built residential areas) were had low-vitality. Effect of the other factors on urban vitality were not evident, indicating that urban vitality may not depend on these factors.

#### 3.4.3. Impact of environmental comfort

Table 5 provides the results of the OLS regression analysis of the impact of comprehensive environmental comfort on urban vitality. For the environmental factors, the goodness-of-fit for the afternoon was better than that for the morning or evening. The *R*^2^ values for the model in the morning, afternoon, and evening were 0.701, 0.726, and 0.671, respectively, and the corresponding regression coefficients of the model were 0.838, 0.852, and 0.819, indicating that comprehensive environmental comfort had a significant positive impact on urban vitality.

**Table 5:** Results of the OLS regression analysis of the impact of comprehensive environmental comfort on urban vitality.

**Urban vitality**	**Comprehensive environmental comfort**	***R* ^2^**	**Adjusted *R*^2^**	**F**
Morning	0.838^**^	0.702	0.701	1648.605763
Afternoon	0.852^**^	0.726	0.726	1864.033411
Evening	0.819^**^	0.670	0.670	1428.776543

^*^Significant at the 0.05 level (two-tailed test).

** Significant at the 0.01 level (two-tailed test).

In terms of the impact of time, the impact of environmental comfort on urban vitality followed the descending order of afternoon> morning> evening. Environmental comfort had a fixed value, and the urban vitality intensity followed the descending order of afternoon> evening> morning, indicating that in the morning and evening, urban vitality was also affected by environmental factors in addition to comprehensive environmental comfort.

### 3.5. Residential and commercial area adjustment and optimization

#### 3.5.1. Residential area

**Figure 7:**
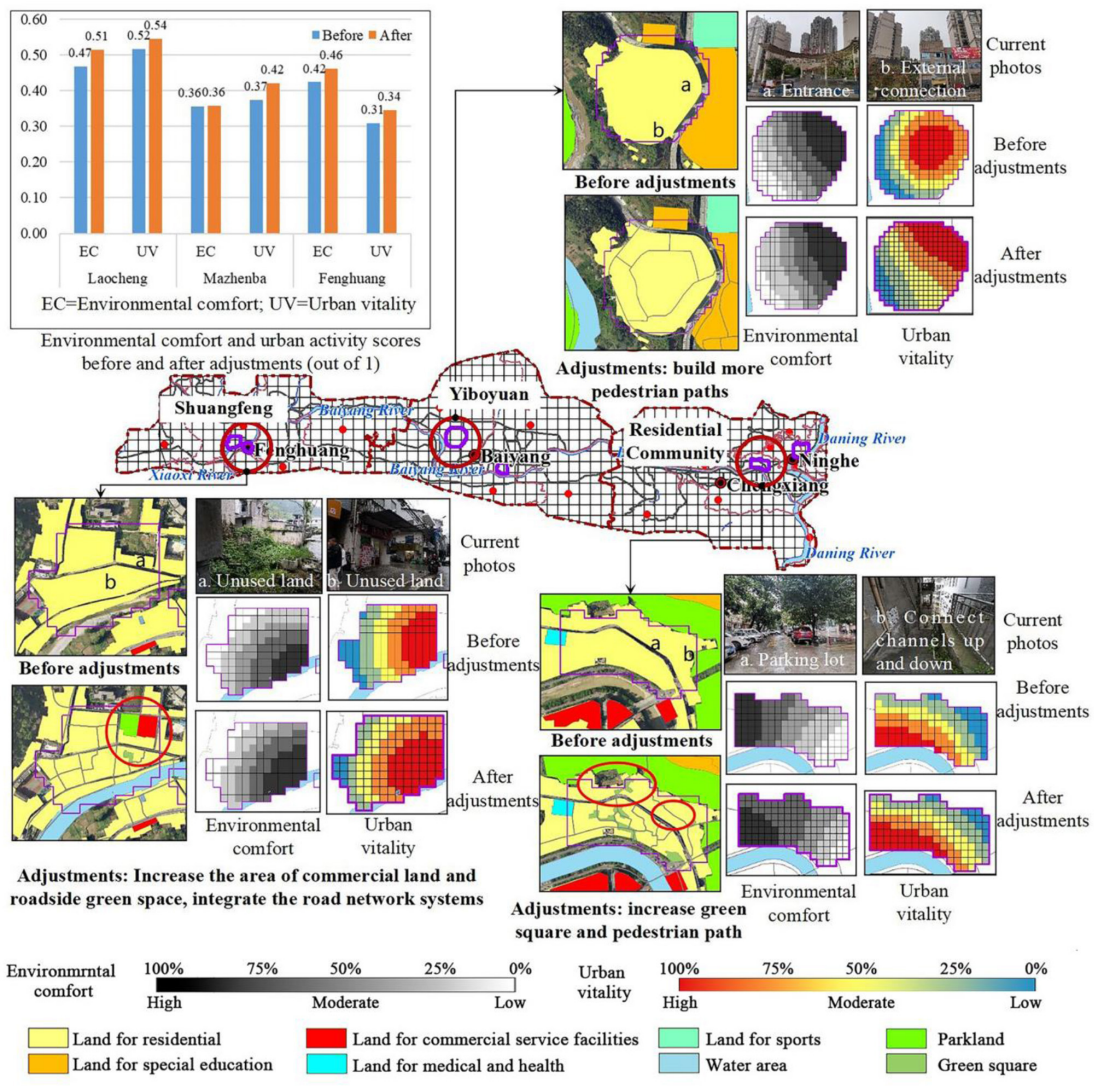
Vitality before and after adjustments of environmental factors in residential areas.

The current situation of the three residential areas was analyzed and optimized in terms of land use type, road network density and public service facilities (Figure 7).

The densely built residential areas of Laocheng have few public spaces, resulting in a unsystematic road network and poor use of existing public spaces. Improving environmental comfort requires not only coordination with the surrounding area, but also attention to the circulation of the system within the system. The public space in Laocheng should be utilized to increase the area of green space and reduce the building density appropriately. Therefore, the specific measure is to integrate the unused public space into small squares within the district. Besides, some old buildings were demolished and integrated to form pedestrian paths, which increased the internal connectivity of residential areas. The adjustments slightly reduced the building density and improved the green coverage. The local road network density and accessibility improved to certain extents. In addition, the average overall environmental comfort increased by 9.70%, and the urban vitality increased by 5.49%.

The new residential area in Mazhenba has relatively low green coverage, low building density, and is a high-rise building. There are more public spaces in the residential area, and the surrounding environment is relatively well equipped. However, due to the closed nature of the district, it is not closely connected with the surrounding areas. A complete pedestrian system should be established to increase the connection with the surrounding areas. Thus, major adjustments mainly occurred with roads. In order to improve the accessibility of residential areas, more pedestrian roads accessible to the outside were built. After adjustments, the local accessibility and road network density improved, the average environmental comfort increased by 0.71% with the average urban vitality increasing by 12.56%.

The residential area of Fenghuang is similar to Laocheng in terms of architecture. The difference is that the surrounding facilities are lacking, and the population is not concentrated due to topographical factors and the different development centers within the clusters, and there are multiple centers in the city. The coverage of public service facilities should be increased, and the internal transportation system should be improved, public green areas should be increased, and the accessibility of residential areas should be improved. In northern Fenghuang, the proportion of commercial land and street green space increased, and the road network systems inside the residential areas were integrated. After adjustments, the average environmental comfort increased by 9.12%, and the average urban vitality increased by 11.77%.

#### 3.5.2. Commercial area

**Figure 8:**
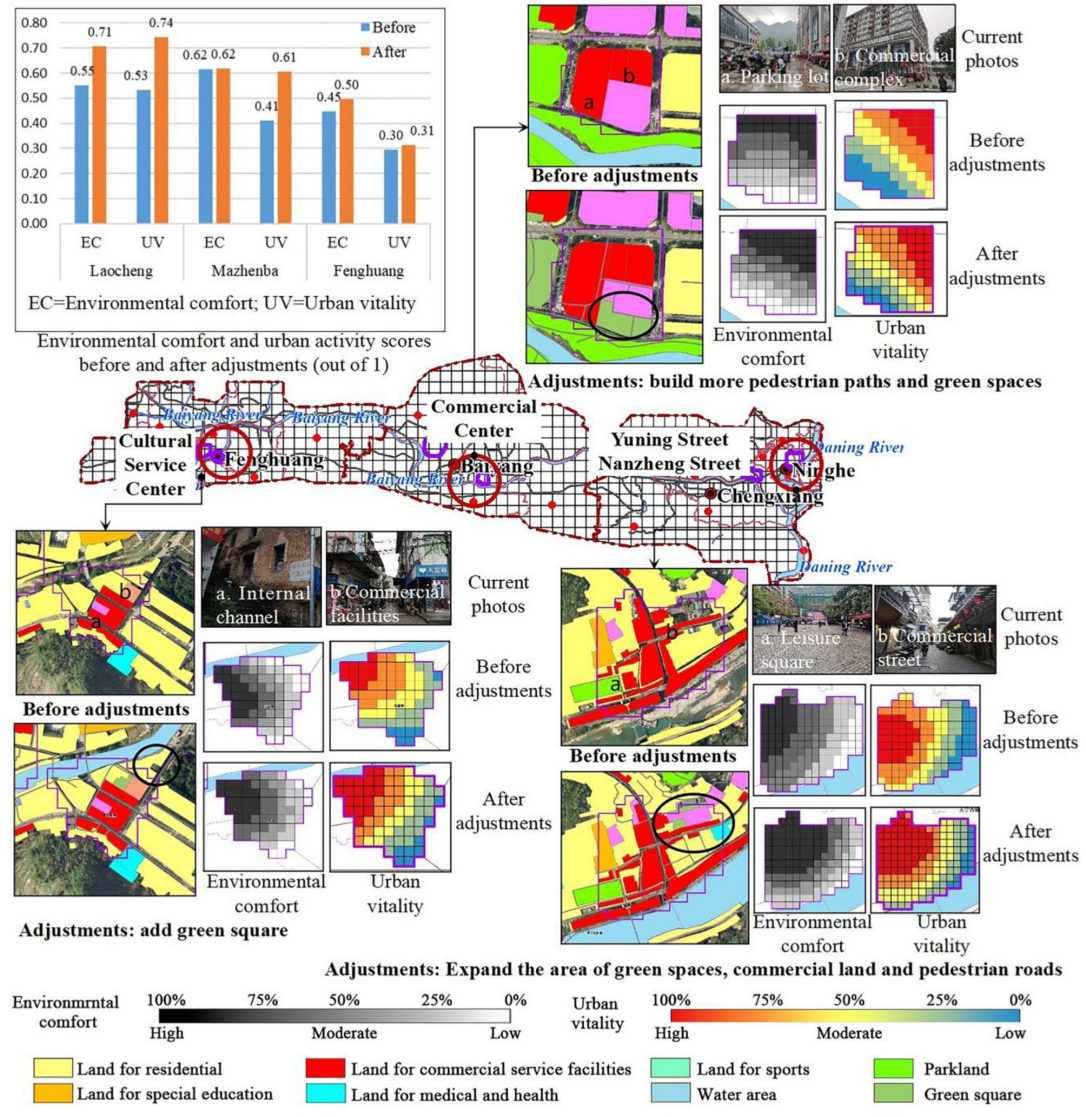
Vitality before and after adjustments of environmental factors in commercial areas.

The current site conditions of the three commercial areas were analyzed and optimized in terms of site type, road network density, and public service facilities, respectively (Figure 8).

The commercial area of Laocheng is one of the high-vitality areas, and although it has a certain scale, it will be interspersed with residential land uses. Take advantage of the mix of current sites to improve the public service facilities support and road network system. Improve accessibility, enhance the flow of people with the surrounding sites, and promote the generation of urban vitality. The commercial areas of Laocheng were combined with unused land in the north. It is supplement to the existing public service facilities and increases the area of green space and commercial land. It promotes the connectivity between the land parcels, and pedestrian paths. After adjustments, the local road network density, accessibility, and also increases density of public service facilities. In addition, the average environmental comfort increased by 28.33% with average vitality increasing by 39.48%.

The center of Mazhenba's business district is modern, with a certain advantage in terms of building age, building density and public services, with a high level of POI mix and integrated accessibility already achieved. The main adjustment was to build more pedestrian paths in Mazhenba. It was recommended that the parking lot for the current administrative office should be adjusted to that on green space or underground. The increase in ground public space will attract more people, thus improving vitality. After the adjustment, the green coverage rate and accessibility both increased, the average environmental comfort increased by 0.15%, and the average urban vitality increased by 47.60%.

The commercial centers in Fenghuang are relatively concentrated, and most of the commercial functions are already embedded in the residential land. Increasing the density of public service facilities and road network in the commercial areas and appropriately renewing the buildings will help improve environmental comfort and urban vitality. Due to the lack of green space, the unused land in the residential areas was adjusted to green space, which increased the green coverage rate and the density of public service facilities. Finally, the average comfort increased by 10.50% along with 6.10% increasing of vitality.

## 4. Discussion

### 4.1. Residential area vitality enhancement strategy

Before adjustment, the environmental comfort in residential areas followed the descending order of Laocheng> Fenghuang> Mazhenba, and the urban vitality followed the descending order of Laocheng> Mazhenba> Fenghuang. Fenghuang had higher environmental comfort but lower urban vitality than Mazhenba. The result indicated that environmental comfort had a positive role in promoting urban vitality. However, in residential areas, the higher the environmental comfort was, the lower the urban vitality. A place with better environmental conditions and basic service facilities is more attractive, but in small and medium-sized cities, few people can afford housing in an excellent environment (which means higher housing prices and basic costs) due to the poor economic conditions. In contrast, the consumption level of places with low environmental comfort can easily matches people's expectation. Therefore, these places attract more people, thereby enhancing urban vitality. In the residential areas of small and medium-sized cities, although environmental comfort has a positive role in promoting urban vitality, higher comfort does not necessarily mean higher vitality.

For residential areas at different stages of development, they should be targeted to improve environmental comfort and urban vitality. Laocheng's residential areas are development saturated neighborhoods, usually with high building density and old age, few public spaces and poor road network systems. Therefore, improving environmental comfort requires not only coordination with the surrounding area, but also attention to the circulation of the system within the system. Reasonable use of the current public space, increase the green space, moderate reduction of a certain amount of building density. The residential areas in the accelerated development period have been built for a short period of time and are not integrated enough with the surrounding environment, and the greening coverage of the newly built neighborhoods is relatively low. However, there are more public spaces in the residential areas and the surrounding environment facilities are relatively perfect. It is necessary to establish a perfect pedestrian system, increase the green coverage, echo the surrounding environment, create excellent environmental comfort, attract crowds, and thus improve urban vitality. The residential areas in the starting period are relatively lacking in various kinds of supporting facilities, polycentric and not concentrated in population in the surrounding area. It is recommended to integrate the surrounding resources, increase the coverage of public service facilities, increase public green areas, improve the internal transportation of residential areas and increase accessibility.

### 4.2. Commercial area vitality enhancement strategy

Before adjustments, the environmental comfort followed the descending order of Mazhenba> Laocheng> Fenghuang, and the urban vitality followed the descending order of Laocheng> Mazhenba> Fenghuang (Figure 8). Mazhenba had higher comfort but lower vitality than Laocheng. The phenomenon indicating that environmental comfort had a positive promoting effect on vitality. In the commercial areas, higher comfort did not necessarily mean higher urban vitality. A place with better environmental conditions and basic service facilities is more attractive, however, newly established commercial centers with superior environmental quality may be less attractive due to their high consumption levels. In contrast, the commercial streets in Laocheng with lower environmental comfort attract more people because their consumption levels meet people's expectations, and naturally, they are more popular.

Commercial areas that have reached the saturation stage of development take advantage of the scale centrality of the existing commercial areas and the mixed scale with other sites. Improve public service facilities support and road network system. Improve accessibility and enhance the flow of people between the site and the surrounding sites. The commercial areas of small and medium-sized cities in the period of accelerated development have the characteristics of commercial centers of modern cities, and the buildings are mostly in the form of complexes. However, the poor comfort of the environment of the chemical outdoor space can lead to a contrast between the indoor and outdoor crowd stopping. It is suggested to strengthen the creation of outdoor public space, extend people's stay in outdoor public space as long as possible, and enhance the urban vitality of outdoor space. Also improve the transportation system within the commercial area to improve accessibility and attract crowds. The commercial area in the initial stage of development has more mixed commercial and residential sites with scattered business forms, making it difficult to form a large-scale and centralized commercial area. Taking into account the situation of the surrounding sites and the advantageous position of the commercial area, more merchants are attracted to move in, the density of public service facilities and road network in the commercial area is increased, and building renewal and the creation of outdoor public space are appropriately carried out.

### 4.3. Analysis of the influence pathways

**Figure 9:**
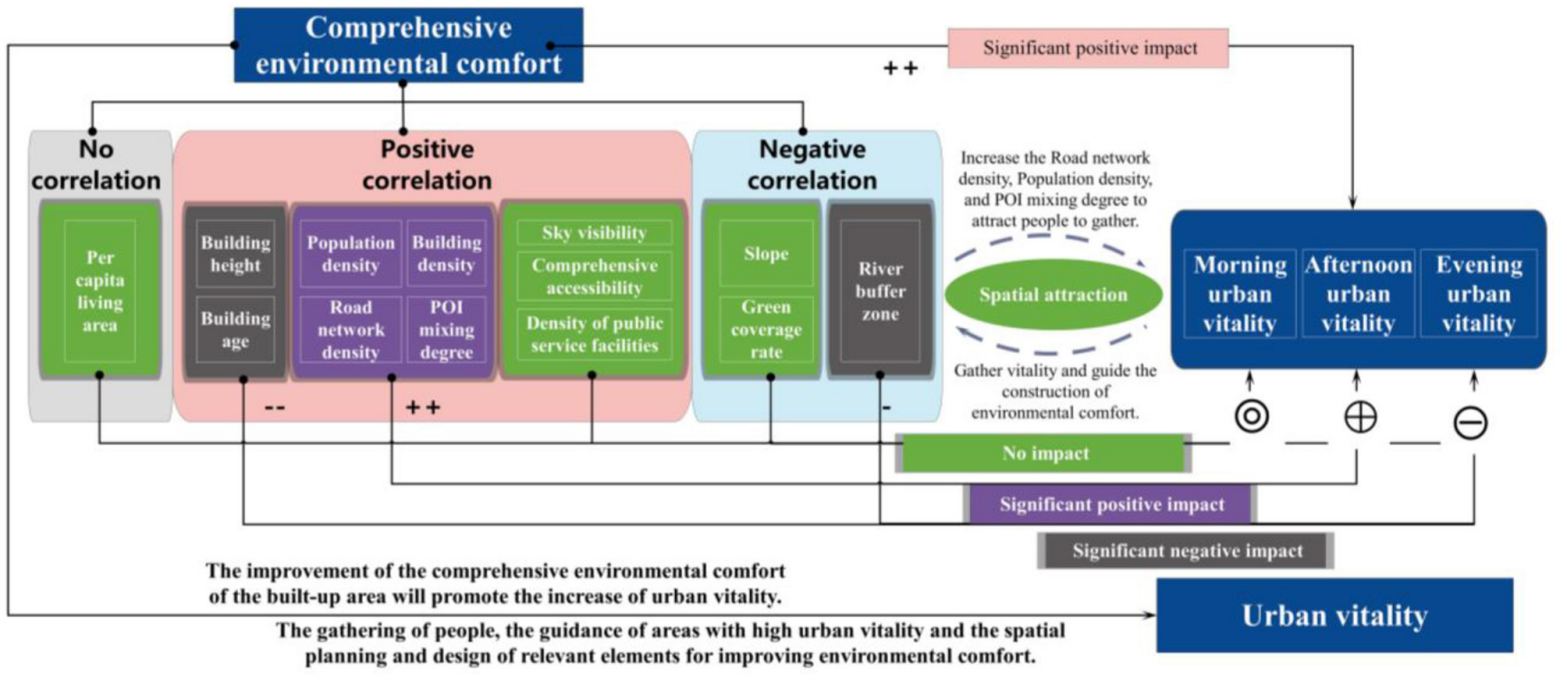
Analysis of the influence pathways in Laocheng.

Take Laocheng as an example. Urban vitality was simultaneously affected by multiple factors (Figure 9), and comprehensive environmental comfort had a significant positive impact on urban vitality.

Among the natural environmental factors, the interiors of the three clusters have a flat terrain and small slopes, and all are suitable for construction. Slopes did not exhibit a regular spatial variation pattern, and their impact on urban vitality was not significant. However, river buffer zones had a significant negative impact on urban vitality. Construction areas concentrated along the Baiyang River, Daning River, and Xiaoxi River. Areas farther away from the river had lower urban vitality. However, the river buffer zones cover a large proportion of the north of Paotai Mountain and north of the Fenghuang community, but the urban vitality was low due to the lack of urban construction activities.

Among the built environment factors, road network density, POI mixing degree, and building density exhibited the same trends as urban vitality. A large proportion of urban area was occupied by roads, and the areas with high POI mixing rates were mainly in the vicinity of Fenghuang Industrial Park and the Fenghuang Community Residential Committee, showing a dual-center pattern. However, urban vitality gradually decreased outward from the Fenghuang Community Residential Committee. Therefore, the specific impact of POI mixing rate cannot be determined. Road network density and urban vitality gradually decreased from the center out. A closer distance to the center, a denser road network, a higher building density, and a higher degree of POI mixing. The buildings in the central area are relatively old and were built in high density, with no significant difference in building height. The vitality there was high. However, the surrounding residential buildings are taller than the buildings in the central area. Hence, high building age and height always negatively impact urban vitality. The green coverage rate, sky visibility, comprehensive accessibility, and public service facilities did not significantly affect urban vitality. The urban areas had relatively high green coverage, high sky visibility, and small spatial heterogeneity, making it difficult to determine the impact of these factors. Accessibility, like road network density, density of public service facilities, and POI mixing degree, may positively impact urban vitality.

In the social environment, population density had a significant positive impact on urban vitality. Areas with higher environmental comfort are more likely to attract people. The central areas had high population densities and high urban vitality. The areas farther away from the urban centers had lower population densities and lower urban vitality. However, the impact of per capita living area was not significant, and the surrounding residential areas were scattered, with no evident centrality. In addition, the per capita living area was high in urban residential areas, but urban vitality was lower than commercial areas.

### 4.4. Limitations

There are still some limitations in the study, which need further exploration. First of all, our research focuses on mountainous cities. The research results can be better applied in mountainous cities, but it may be different from the construction of plain cities that are not affected by factors such as terrain. For other mountainous cities, the selection of environmental elements should also be adjusted according to local conditions to obtain more accurate results and apply them to the improvement of local environmental comfort. In the survey of plain cities, we can consider adding big data and spatial-temporal quantitative analysis, and adding more subjective satisfaction surveys. Second, the urban vitality investigated is mainly represented by Tencent Easygo heat map, but there are different interpretations of urban vitality, which should be further optimized. Then, although there are many types involved in environmental comfort elements, all the factors affecting environmental comfort still cannot be included. The factors of environmental comfort need to be improved in further study. Last but not least, the method used in the study involves multiple software and data processing, so there may be some problems in inter-technical parameter settings, and optimization is needed in the future.

## 5. Conclusion

Multiple data sources were used to analyze the impact of environmental comfort on urban vitality as well as their spatial autocorrelation and spatial matching. It was found that:

(1) Environmental comfort is significantly positively correlated with urban vitality. The environmental comfort and urban vitality of the three groups all present a single-center pattern. Environmental comfort and urban vitality decrease from the city center to the periphery, and gradually decrease from east to west as a whole. It is high in the east and low in the west, high in the south and low in the north.

(2) Urban vitality is significantly influenced favorably by population density, POI mixing percentage, building density, and road network density. Population density has the greatest impact on urban vitality, followed by road network density. The influence of POI mixing degree on urban vitality is greatest in the afternoon, and the influence of building density is greatest in the morning. Building height, building age, and river buffer have significant negative effects on urban vitality, indicating that buildings that are taller, newer, and farther from the river reduce urban vitality.

(3) Comprehensive environmental comfort has a significant positive impact on urban vitality. In terms of time, the influence of comprehensive environmental comfort on urban vitality in descending order is afternoon > morning > evening.

By looking for the matching degree between environmental comfort and urban activities, this study discovered the main environmental factors that affect urban vitality and finally constructed an evaluation method for the impact of environmental comfort on urban vitality. Although population density and building density can promote urban vitality, excessive population density and building density can cause damage to people's physiology and psychology. In the development of the urban built environment, attention should be paid to the balance between the needs of developing cities in different periods and with different characteristics to meet the needs of improving urban vitality at different levels. When the development of small and medium-sized cities reaches saturation, they should pay attention to the evacuation of the central area and improve the comfort of the environment outward. When the development is accelerated, use the central area to drive the synchronous development of the surrounding areas. When the development is in its infancy, strengthen the construction of the central area.

In residential areas, we recommend that during the period of saturated development, the current public space be utilized judiciously, green spaces be increased, and building density be appropriately reduced. During the accelerated development period, establish a complete pedestrian system to improve the connection with the surrounding area. To develop residential areas in the initial stage, integrate surrounding resources, increase the coverage of public service facilities, increase public green spaces, improve internal traffic in residential areas, and improve accessibility. For the commercial districts, to increase population mobility and encourage the creation of urban vitality, public service facilities and the road network system should be improved once development has reached saturation. During the accelerated development period, indoor and outdoor spaces' attractiveness, the length of time people spends in public areas, the internal transportation system, accessibility, and the creation of a top-notch shopping environment should all be improved. Improve the transportation system while introducing commercial and public service facilities during the early stages of development.

Overall, our findings and evaluation methods can promote large improvements in vitality under minor adjustment. It is of great significance on urban spatial planning and promotion of new urbanization and rural revitalization. Meanwhile, it can provide reference for planning and design in small and medium-sized cities.

## Data availability statement

The data analyzed in this study is subject to the following licenses/restrictions: The raw data supporting the conclusions of this article will be made available by the authors, without undue reservation. Requests to access these datasets should be directed to hangf@cqu.edu.cn.

## Author contributions

GL: conceptualization, formal analysis, investigation, data curation, and writing—original draft. JLe: formal analysis, resources, and writing—review and editing. HQ, JN, and JC: writing—reviewing and editing. JLu: resources and writing—review and editing. GH: conceptualization, methodology, funding acquisition, supervision, and writing—review and editing. All authors contributed to the article and approved the submitted version.

## References

[1] United Nations. 2018 Revision of world urbanization prospects. New York: United Nations. (2018). Available online at: https://esa.un.org/unpd/wup/ (accessed July 8, 2021).

[2] Gu C. Urbanization: processes and driving forces. Sci China Earth Sci. (2019) 62:1351–60. 10.1007/s11430-018-9359-y

[3] Sun L Chen J Li Q Huang D. Dramatic uneven urbanization of large cities throughout the world in recent decades. Nat Commun. (2020) 11:5366. 10.1038/s41467-020-19158-1 33097712 PMC7584620

[4] National Bureau of Statistics of China. Statistical Communiqué of the People's Republic of China on the 2021 National Economic and Social Development. (2022). Available online at: http://www.stats.gov.cn/index.html (accessed April 6, 2022).

[5] Wang Y Wang J Liu Y Li J. Calibrations of urbanization level in China. China CDC Wkly. (2022) 4:111–5. 10.46234/ccdcw2022.007 35186381 PMC8844521

[6] Zhang Z. Travel Mode Experience Design and Research in Post-Urbanization Era (Master's Thesis). Tianjin: Tianjin Academy of Fine Arts (2022).

[7] Meng P. Research on the Optimum of the Development of Urbanization: A Case Study in Huang-Huai-Hai Plain (Doctoral Thesis). Beijing: China Agricultural University (2014).

[8] Luo X Lu J. New development trends of the 14th five-year plan period and the spatial planning response. City Plann Rev. (2019) 43:9–12. 10.11819/cpr20191003a

[9] Huang Y. Space planning: Guiding the balanced urban development. Resour. Guide. (2020) 11:20–1. 10.3969/j.issn.1674-053X.2020.11.016

[10] Rong P Zhang L Qin Y Xie Z Li Y. Spatial differentiation of daily travel carbon emissions in small-and medium-sized cities: an empirical study in Kaifeng, China. J Clean Prod. (2018) 197:1365–73. 10.1016/j.jclepro.2018.06.205

[11] Qi Y Wu J Li J Yu Y Peng F Sun C. Landscape dynamics of medium-and small-sized cities in eastern and western China: a comparative study of pattern and driving forces. Shengtai Xuebao/Acta Ecologica Sinica. (2013) 33:275–85. 10.5846/stxb201111091694

[12] Terjung WH. Physiologic climates of the conterminous United States: a bioclimatic classification based on man. Ann Assoc Am Geogr. (1966) 56:141–79. 10.1111/j.1467-8306.1966.tb00549.x

[13] Ren X Li Y Wang J. Evaluation of climate comfortability for human settlement environment in northern coastal cities in recent 60 years: taking Liaoning as a case. J Nat Resour. (2013) 28:811–21. Available online at: https://kns.cnki.net/kcms2/article/abstract?v=3uoqIhG8C44YLTlOAiTRKgchrJ08w1e7eWoVfj7plMwFppTmeNj_s8pYCAwloGlTwreqxTsMm5he6P69TnIXb-mOskzQFHsU&uniplatform=NZKPT

[14] Xiang Z Qin H He B Han G Chen M. Heat vulnerability caused by physical and social conditions in a mountainous megacity of Chongqing, China. Sustain Cities Soc. (2022) 80:103792. 10.1016/j.scs.2022.103792

[15] Qin H Cheng X Han G Wang Y Deng J Yang Y. How thermal conditions affect the spatial-temporal distribution of visitors in urban parks: a case study in Chongqing, China. Urban Fort Urban Green. (2021) 66:127393. 10.1016/j.ufug.2021.127393

[16] Su H Han G Li L Qin H. The impact of macro-scale urban form on land surface temperature: an empirical study based on climate zone, urban size and industrial structure in China. Sustain Cities Soc. (2021) 74:103217. 10.1016/j.scs.2021.103217

[17] Guo J Han G Xie Y Cai Z Zhao Y. Exploring the relationships between urban spatial form factors and land surface temperature in mountainous area: a case study in Chongqing city, China. Sustain Cities Soc. (2020) 61:102286. 10.1016/j.scs.2020.102286

[18] Yan J. Research on Design Strategy of the Outdoor Space of Block-Style Commercial Complex Based On Thermal Comfort (Master's Thesis). Harbin: Harbin Institute of Technology (2020).

[19] Cao B Zhu Y Ouyang Q. The relationship between indoor environmental quality of public buildings and human body comfort. Build Sci. (2010) 26:126–30. 10.13614/j.cnki.11-1962/tu.2010.10.027

[20] Si Y Yu J Wang N Huang J Ding X. Indoor comfort assessment of large public building based on sustainability key performance indicators. In: The 11th International Conference on Green and Energy-Efficient Building & New Technologies and Products Expo. Beijing (2015). p. 92–7 (in Chinese).

[21] Zhao Z Sharifi A Dong X Shen L He BJ. Spatial variability and temporal heterogeneity of surface urban heat island patterns and the suitability of local climate zones for land surface temperature characterization. Remote Sens. (2021) 13:4338. 10.3390/rs13214338

[22] He B-J Wang J Zhu J Qi J. Beating the urban heat: Situation, background, impacts, and the way forward in China. Renew Sustain Energy Rev. (2022) 161:112350. 10.1016/j.rser.2022.112350

[23] Nichols Clark T. 3. Urban amenities: lakes, opera, and juice bars: do they drive development? City Enter Mach. (2003) 9:103–40. 10.1016/S1479-3520(03)09003-2

[24] Brown WM Scott DM. Cities and growth: Human capital location choice: accounting for amenities and thick labor markets. Can Econ Trans Res Paper. (2012) 27:1853. 10.2139/ssrn.2141853

[25] Dang Y Yu J Zhang W. Satisfaction evaluation of living environment and influencing factors in the Bohai Rim area. Prog Geogr. (2016) 35:184–94. 10.18306/dlkxjz.2016.02.005

[26] Yun Y Yu Y. How to construct and evaluate commercial pedestrian streets' comfort. Urban Dev Stud. (2008) 15:36–42. 10.3969/j.issn.1006-3862.2008.03.014

[27] Wang Y Liu X Pan X. Study on the vitality source of Shanghai in the future. Scientif Dev. (2017) 2017:5–14. 10.3969/j.issn.1674-6171.2017.09.001

[28] Jane J. The death and life of great American cities. Sci Cult. (2006) (7):56–7. Available online at: https://kns.cnki.net/kcms2/article/abstract?v=3uoqIhG8C44YLTlOAiTRKgchrJ08w1e7eWoVfj7plMwFppTmeNj_s8pYCAwloGlTwreqxTsMm5he6P69TnIXb-mOskzQFHsU&uniplatform=NZKPT

[29] Lewis M. The City in History: Its Origins, Its Transformations, and Its Prospects. New York: Houghton Mifflin Harcourt (1961).

[30] Mi Y. The Vigor Construction Policy of City Street Based on Public Behavior Survey (Master's Thesis). Changsha: Central South University (2014).

[31] Ye Y Zhuang Y Zhang L van Nes A. Designing urban spatial vitality from morphological perspective: a study based on quantified urban morphology and activities' testing. Urban Plan Int. (2016) 31:26–33.

[32] Zhou M. Study on the Outside Space's Vitality of City Commercial Center District: The Example of Guanyinqiao Commercial Center District in Chongqing City (Master's Thesis). Chongqing: Chongqing University (2007). 10.7666/d.y1139001

[33] Katz P. The New Urbanism: Toward an Architecture of Community. New York: McGraw-Hill (1994).

[34] Lu F Xu Y. Rebuilding the landscape factors in the designs of mountainous-waterfront city: the example of Chongqing city. Chin Landsc Architect. (2006) 2006:61–4. 10.3969/j.issn.1000-6664.2006.06.013

[35] Weng S. Analysis of Urban Vitality for Wuhan: A Case Study in Main City Zone (Master's Thesis). Wuhan: Wuhan University, (2019).

[36] Chen L Ng E. Outdoor thermal comfort and outdoor activities: a review of research in the past decade. Cities. (2012) 29:118–25. 10.1016/j.cities.2011.08.006

[37] Zhang S. Study on Thermal Comfort of Shaded Environment Within Urban Green Open Space in Wuhan (Master's Thesis). Hangzhou: Huazhong Agricultural University (2018).

[38] Işiklar S Kirci N. Assessment of Selânik Street/Ankara as a living urban space. IOP Conf Ser: Mater Sci Eng. (2017) 245:72008. 10.1088/1757-899X/245/7/072008

[39] Lai D Chen B Liu K. Quantification of the influence of thermal comfort and life patterns on outdoor space activities. Build Simulat. (2020) 13:113–25. 10.1007/s12273-019-0565-x

[40] Qi L. Statistics analysis and fuzzy comprehensive evaluation of Likert scale. Shandong Sci. (2006) 19:18–23. 10.3969/j.issn.1002-4026.2006.02.006

[41] Lin J He J Huang X. Research on the city preference of the flow of scientific research talents from the perspective of urban amenities. Areal Research Dev. (2020) 39:59–64.

[42] Cao W Wang S. Evaluation of climate suitability for urban human settlement in Beijing-Tianjin-Hebei region. J Glaciol Geocryol. (2017) 39:435–42. 10.7522/j.issn.1000-0240.2017.0050

[43] Yang Q. Study on Evaluation and Renewal Strategy of Environmental Livability in Old Residential Quarters in Chengdu: Take Renbei Area as an Example (Master's Thesis). Chengdu: Southwest Jiaotong University (2018).

[44] Bao H. Comfort Evaluation and Spatial Heterogeneity Analysis of Livable Cultural Environment in Beijing (Master's Thesis). Beijing: Capital Normal University (2011).

[45] Rong X Wang M. The residential comfort evaluation for living areas of Xuzhou city. Value Engineering. (2013) 32:312–4. 10.14018/j.cnki.cn13-1085/n.2013.07.070

[46] Guo H Guo Y Cui N. Research on typical population aggregation districts in the Beijing's sixth ring road based on Baidu's heat map and points of interest. Urban Dev Stud. (2018) 25:107–12. 10.3969/j.issn.1006-3862.2018.12.020

